# Does anti-VEGF bevacizumab improve survival in experimental sepsis?

**DOI:** 10.1186/s13054-017-1734-x

**Published:** 2017-07-05

**Authors:** Emmanuel Besnier, Ebba Brakenhielm, Vincent Richard, Fabienne Tamion

**Affiliations:** 1 Normandie Univ, UNIROUEN, INSERM U1096, 76000 Rouen, France; 2 Department of Anaesthesia and Critical Care, Rouen University Hospital, 76000 Rouen, France; 3 Medical Critical Care Unit, Rouen University Hospital, 76000 Rouen, France

In a previous issue of *Critical Care*, Jeong et al. reported a beneficial effect of bevacizumab (Bev), the first humanized vascular endothelial growth factor (VEGF)-neutralizing antibody, on vascular permeability and mortality in a murine model of sepsis [1]. VEGF has been associated with mortality during sepsis [2], and the administration of its natural antagonist improved survival in experimental sepsis [3]. Jeong et al. demonstrated that Bev reduced mortality in sepsis induced either by cecal ligature puncture (CLP) or by endotoxemia. Despite promising experimental data, no other study has yet confirmed these results. A clinical study was planned to evaluate Bev administration in critically ill patients but it was withdrawn before enrolment (NCT01063010).

Thus, we aimed to reassess the potential benefit of Bev during experimental sepsis. After approval by the *Haute-Normandie* ethics committee (number 8092), male C57Bl6 mice received intraperitoneal NaCl (control) or Bev (0.5 mg/kg) immediately before CLP (*n* = 15/group), performed as described previously [4]. Briefly, the cecum was ligated (75% of total length) and punctioned bilaterally with a 21G needle. Topical lidocaïne (2%) was applied and mice received a sub-cutaneous administration of the antibiotic ofloxacine (30 mg/kg), the analgesic tramadol (40 mg/kg), and NaCl (30 ml/kg). Survival was evaluated twice per day for 10 days and analyzed through a log-rank test.

No significant difference in mortality was observed between the Bev and control groups (36 versus 27% at day 10, *p* = 0.64). To overcome any non-optimal effect linked to the route of administration, and also to better mimic clinical use, we repeated the experiments with intravenous injection of Bev before surgery (*n* = 8/group). Again, we did not observe any effect on mortality compared to CLP controls (42 versus 37% at day 10, *p* = 0.74). Pooling the data between experiments (Bev treatment either IP or IV versus controls; *n* = 23/group) also did not show any statistical difference (38 versus 31%, *p* = 0.56; Fig. 1).

**Fig. 1:**
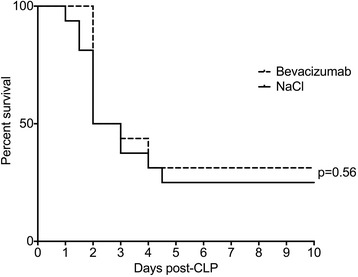
Survival of septic mice treated with bevacizumab or NaCl over a ten days period

Even if our experimental procedure slightly varies, notably regarding the severity of sepsis, with a larger puncture site, and the use of intravenous route in some of the mice, these results contradict those described by Jeong et al. The absence of replication of their results may be surprising, notably regarding the suspected effects of the VEGF pathways in sepsis. Although we cannot identify the origin of this contradiction, the absence of new publications on this topic, in association with our negative results, raises the question of the clinical rational of anti-VEGF treatment in septic patients.

## Abbreviations

Bev: Bevacizumab
CLP: Caecal ligation and puncture
VEGF: Vascular endothelial growth factor
